# CLEAN: CLustering Enrichment ANalysis

**DOI:** 10.1186/1471-2105-10-234

**Published:** 2009-07-29

**Authors:** Johannes M Freudenberg, Vineet K Joshi, Zhen Hu, Mario Medvedovic

**Affiliations:** 1 Laboratory for Statistical Genomics and Systems Biology, Department of Environmental Health, University of Cincinnati College of Medicine, 3223 Eden Av. ML 56, Cincinnati OH 45267-0056, USA

## Abstract

**Background:**

Integration of biological knowledge encoded in various lists of functionally related genes has become one of the most important aspects of analyzing genome-wide functional genomics data. In the context of cluster analysis, functional coherence of clusters established through such analyses have been used to identify biologically meaningful clusters, compare clustering algorithms and identify biological pathways associated with the biological process under investigation.

**Results:**

We developed a computational framework for analytically and visually integrating knowledge-based functional categories with the cluster analysis of genomics data. The framework is based on the simple, conceptually appealing, and biologically interpretable gene-specific functional coherence score (CLEAN score). The score is derived by correlating the clustering structure as a whole with functional categories of interest. We directly demonstrate that integrating biological knowledge in this way improves the reproducibility of conclusions derived from cluster analysis. The CLEAN score differentiates between the levels of functional coherence for genes within the same cluster based on their membership in enriched functional categories. We show that this aspect results in higher reproducibility across independent datasets and produces more informative genes for distinguishing different sample types than the scores based on the traditional cluster-wide analysis. We also demonstrate the utility of the CLEAN framework in comparing clusterings produced by different algorithms. CLEAN was implemented as an add-on R package and can be downloaded at . The package integrates routines for calculating gene specific functional coherence scores and the open source interactive Java-based viewer Functional TreeView (FTreeView).

**Conclusion:**

Our results indicate that using the gene-specific functional coherence score improves the reproducibility of the conclusions made about clusters of co-expressed genes over using the traditional cluster-wide scores. Using gene-specific coherence scores also simplifies the comparisons of clusterings produced by different clustering algorithms and provides a simple tool for selecting genes with a "functionally coherent" expression profile.

## Background

Identifying groups of co-expressed genes through cluster analysis has been successfully used to elucidate affected biological pathways and postulate transcriptional regulatory mechanisms [1,2]. The integration of biological knowledge in such analyses has been most commonly facilitated by assessing the enrichment of clusters with genes from pre-defined functionally coherent gene lists ("functional categories"). The concept of "functionally related genes clustering together" has been established by ad-hoc visual examination of hierarchical clustering results and their enrichment by genes from the same functional category [3]. The first assessment of statistical significance of such enrichments was performed by analyzing results of *k*-means clustering [4] using the hypergeometric distribution [5]. Similar strategies have also been used in the analysis of lists of differentially expressed genes [6], gene lists constructed based on genome-wide Chromatin Immunoprecipitation (ChIP) [7,8] and epigenomics experiments [9], as well as the general approach to integrate lists of genes derived by various experimental and knowledge-based procedures [10]. Introducing biological knowledge through such post-hoc analysis has been important for interpreting results and separating reproducible, biologically meaningful gene clusters from clusters that may have resulted from random fluctuations in the data. For both of these objectives, reproducibility of conclusions made is of utmost importance.

The first two concept defining papers [3,5] also highlight the dichotomy that exist in using hierarchical vs. partitioning clustering procedures to this days. Hierarchical procedures do not necessitate specifying the "right" number of clusters, a parameter generally unknown in advance whose estimation from the data leads to instability in clustering results [11]. On the other hand, selecting "meaningful" clusters in a hierarchical clustering that can be then correlated with functional categories using the hypergeometric distribution is still mostly performed by ad-hoc visual inspection of related heatmaps. Algorithms for systematic testing of all possible clusters have also been developed [12-14], but results of such analyses are difficult to summarize. Postulating the "right" number of clusters or choosing "good" clusters in an ad-hoc fashion before correlating them with functional categories can result in poor reproducibility since a slightly different number of clusters or slightly different "good" clusters can result in a different interpretation of the results. This problem is akin to choosing the "optimal" cut-off criteria for selecting differentially expressed genes before performing similar functional analyses. It has been shown that results of such analyses are highly sensitive to changes in the cut-off used with different cut-offs yielding different conclusions [15]. In the analysis of differentially expressed genes computational alternatives have been developed that do not require setting such thresholds [16-18], but they are generally not applicable in the knowledge-based assessment of clustering results.

A frequently encountered problem in analyzing genome-wide experimental data is to choose among results produced by different clustering algorithms. Criteria such as homogeneity and separation are relatively straightforward to compute but are mostly of theoretical interest. A more relevant criterion from a biological perspective is the overall functional coherence of resulting gene clusters. Most of the methods developed to date for this purpose require specification of the number of clusters [19,20]. Comparing different methods at a fixed number of clusters is problematic as some methods might create a better clustering structure when more clusters are allowed and others could create better clusterings when few clusters are allowed. To circumvent this problem ROC curves have been used to assess false and true positive rates of co-clustered gene pairs using the functional categories as a gold standard[21,22]. However, this same strategy lacked discriminative power when a large number of large functional categories, such as Gene Ontology (GO) terms, served as a gold standard and required again fixing the number of clusters [21,23].

We developed an analytical framework and flexible computational infrastructure for integrating knowledge-based functional categories into the cluster analysis of gene expression data. The framework is based on the simple, conceptually appealing and biologically interpretable gene-specific functional coherence CLustering Enrichment ANalysis (CLEAN) score derived by correlating the clustering structure as a whole with functional categories of interest. The CLEAN score is gene-specific and it differentiates between the levels of functional coherence for genes within the same cluster. The statistical significance of coherence scores is established by comparing them to the empirical null-distribution obtained by randomly permuting gene identifiers. The corresponding computational infrastructure is based on an open-source R package for the data analysis and open-source Java viewer for visually integrating and analyzing expression data and associated knowledge-based functional categories.

We investigate the reproducibility of the findings based on the CLEAN scores, and demonstrate its utility in comparing the functional coherence of clusterings produced by different algorithms and in selecting genes with informative expression patterns. Being gene-specific, the CLEAN score facilitates easy comparisons of functional coherence of different hierarchical structures (e.g. generated by different clustering algorithms) and selection of genes based on functional coherence of their expression pattern without the need to fix the number of clusters. On the other hand, we demonstrate that differentiating between the levels of functional coherence for genes within the same cluster leads to significant improvements in reproducibility of findings across independent microarray datasets when compared to traditional cluster-wide analyses. Furthermore, genes selected based on the CLEAN score produced more precise sample groupings than genes selected using the cluster-wide score.

## Results

**Figure 1:**
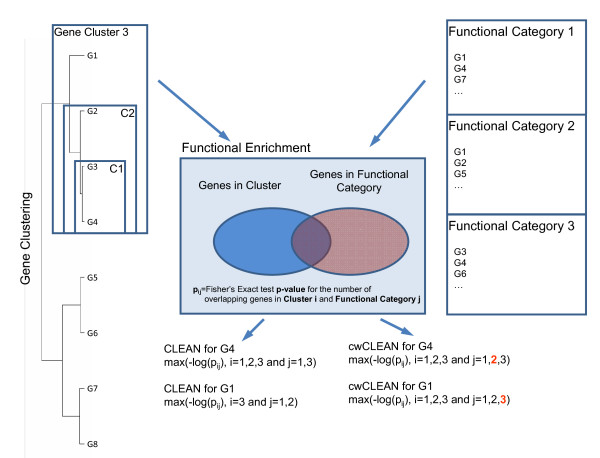
**Calculating functional coherence scores**. Given a hierarchical clustering of genes based on their expression profiles and a set of functional categories (e.g. Gene Ontologies), the CLustering Enrichment ANalysis (CLEAN) score for a gene is calculated as the maximum of -log(Fisher's Exact Test *q*-value) of enrichment tests across all pairs of clusters containing the gene and functional categories containing the gene (see methods for details). The Cluster-wide CLEAN score (cwCLEAN) is calculated in a similar fashion except that the maximum is taken over all clusters that contain the gene and all functional categories regardless of whether they contain the gene or not.

Given a hierarchical clustering of genes based on their expression profiles and a set of functional categories (e.g. Gene Ontologies), the CLustering Enrichment ANalysis (CLEAN) score for a gene is calculated as follows (Figure 1):

1. Fisher's exact test for enrichment is calculated for all functional categories containing the gene and for all possible clusters containing this gene. (Figure 1).

2. The CLEAN score is then computed as the maximum -log_10_(*q*-value) of enrichment tests across all pairs of clusters containing the gene and functional categories containing the gene (see methods for details).

The clustering-specific null-distribution of the CLEAN score is established by randomly permuting gene identifiers. Statistically significant scores are then used to facilitate selection of genes or gene clusters, as well as the assessment of functional coherence and the comparison of clustering results produced by different algorithms. The integrated clustering viewer/browser, Functional TreeView (FTreeView), is used for integrative browsing and visual display of gene clusters and associated functional categories.

When multiple category types are used, the joint CLEAN score is calculated as the maximum of CLEAN scores for each category type. Here we focus on three sets of functional categories: Gene Ontology (GO) categories [24], Kyoto Encyclopedia of Genes and Genomes (KEGG) pathways [25,26], and a custom set of Co-regulation Groups (CG) based on the computational analysis of gene promoters and regulatory motif definitions in the Transfac database, version 12.1 [27] (see methods).

All currently used algorithms assign statistical significance of functional enrichment to whole clusters instead of individual genes within the cluster. To compare the properties of the CLEAN score to currently used methods we define a cluster-wide CLEAN (cwCLEAN) score to serve as a surrogate for this traditional type of analysis. The cwCLEAN score is defined as the maximum of -log_10_(*q*-value) for all clusters containing the gene regardless of whether the enriched functional categories contain the gene or not (Figure 1). We analyzed several public microarray datasets to demonstrate the statistical properties and utility of the CLEAN framework, and to compare its performance to traditionally used approaches.

### Comparing clustering results using the CLEAN score

The CLEAN score provides a tool to compare the functional coherence of clustering results produced by different clustering algorithms on a gene-by-gene basis without requiring a pre-defined number of gene clusters. We used four independent large-scale breast cancer gene expression datasets [28-31] to demonstrate utility of the CLEAN score in choosing the clustering structure with the highest functional coherence. For each dataset we compared the performance of three typical clustering algorithms: Context specific infinite mixture model (CSIMM) [22], Euclidian distance based and Pearson's correlation based hierarchical clustering. For all three algorithms, the hierarchical clustering was constructed using the average linkage principle and algorithms were applied to expression data with and without prior variance-rescaling (see methods). Clustering algorithms were used to cluster data from four independent breast cancer gene expression datasets with GEO accession numbers GSE1456 [29], GSE3494 [28], GSE7390 [31], and GSE11121 [30].

**Figure 2:**
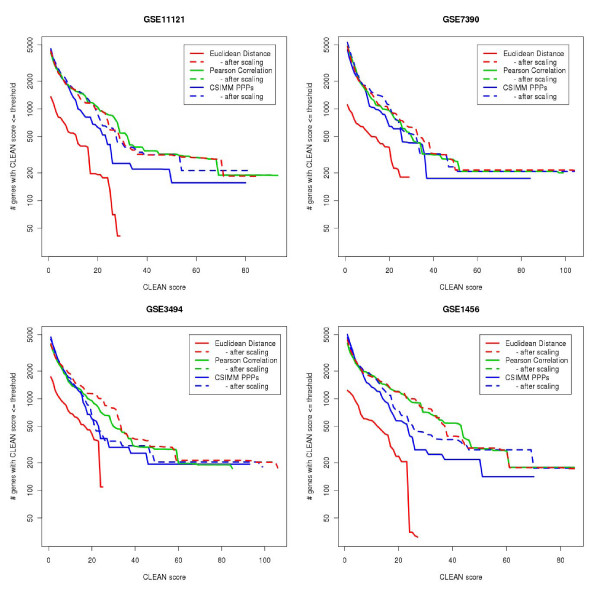
**Comparison of clustering methods**. We compared functional coherence of six clustering algorithms: Context specific infinite mixture model (CSIMM), Euclidian distance based and Pearson's correlation based hierarchical clustering with and without prior variance-rescaling of the data, across four independent human breast cancer datasets (GEO expression series GSE1456 [29], GSE3494 [28], GSE7390 [30], and GSE11121 [31]). For all six algorithms, the hierarchical clustering was constructed using the average linkage principle. The number of genes common in all four datasets after filtering was 6,150. CLEAN scores are plotted against the x-axis and the corresponding number of genes with the CLEAN greater than this are plotted against the y-axis. Higher areas under the curve imply the higher functional coherence.

For each clustering algorithm the total number of genes (y-axis) with the CLEAN score higher than the given threshold was plotted against all possible threshold levels (x-axis). There are two conclusions that can be immediately made based on results in Figure 2. First, variance-based rescaling of the data significantly improved the functional coherence of resulting clustering. While the CSIMM model is capable of compensating this effect to some extent, the performance of both CSIMM and Euclidean distance based algorithms improved after data was re-scaled. Since the Pearson's correlation coefficient implicitly performs such re-scaling, there is little difference between its performance with and without re-scaling. After the data was re-scaled, all three algorithms perform almost identically indicating that the re-scaling is the key step in improving the functional coherence of the data. The second conclusion is that these non-trivial results are perfectly reproducible across four independent breast cancer datasets, which is an important indication about their applicability to other datasets of this type.

### Reproducibility and the comparison with cluster-wide scores

**Figure 3:**
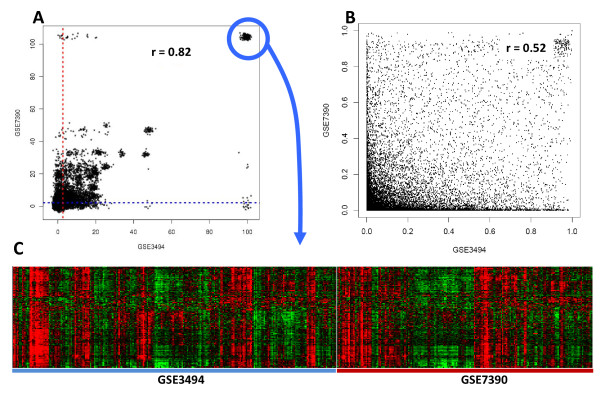
**Integrating cluster analysis and functional knowledge**. Genes were clustered using the CSIMM [22] algorithm and variance-scaled data from two independent breast cancer datasets (GSE3494 [28] and GSE7390 [31]), and CLEAN scores were computed for both clusterings. The number of genes common in both datasets after filtering was 8,567. A) The gene-specific CLEAN scores for the two datasets were plotted against each other and the Pearson's correlation coefficient was computed. A small error was added in the scatter plot to better visualize overlapping data points. B) Pairwise similarity measures between genes computed by CSIMM were also plotted and correlated. C) Expression profiles of genes with the very highest CLEAN scores in both datasets showed strong co-expression in both datasets. All genes in this cluster are immunity related.

We used the same four independent breast cancer gene expression datasets (GSE1456 [29], GSE3494 [28], GSE7390 [31], and GSE11121 [30]) and a study comparing tissue-specific gene expression patterns in mouse and human [32] to investigate reproducibility of the CLEAN scores. We first assessed the contribution of the functional data to the reproducibility of the clustering results by comparing the correlation between the CLEAN scores (Figure 3A) to correlation of pair-wise distances used to construct the hierarchical clustering of genes (Figure 3B) in the two datasets (GSE3494 and GSE7390). In this analysis pairwise distances are based on the Bayesian posterior pairwise probabilities (PPPs) produced by the CSIMM algorithm [22]. Significantly increased correlation for the CLEAN sore (0.82 vs. 0.52 PPPs) indicated a significant increase in reproducibility of results in terms of functional coherence of the gene expression patterns over the simple clustering that does not incorporate an assessment of functional coherence. The heatmap of expression profiles for the genes with the highest CLEAN scores in both datasets (circled in the Figure 3A) showed a coherent pattern of expression within both datasets (Figure 3C) and all these genes are related to immune system, which is a commonly implicated functional group in the etiology of cancer in general.

**Figure 4:**
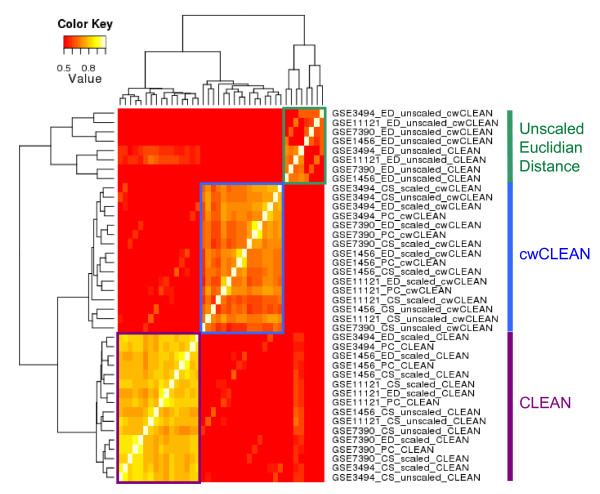
**Reproducibility of CLEAN and cwCLEAN scores**. The reproducibility of the functional coherence results for 6 different clustering algorithms was assessed by calculating all pairwise Pearson's correlation coefficients between scores for all algorithms applied to four independent human breast cancer datasets (GEO expression series GSE1456 [29], GSE3494 [28], GSE7390 [31], and GSE11121 [30]). Rows and columns in this symmetric heatmap represent specific scores for a specific clustering in a specific dataset in the heatmap. The symmetric hierarchical clustering of rows and columns was constructed using pairwise Pearson's correlations between different scores as the similarity measures and applying the complete linkage principle.

Next, we performed a comprehensive study of the reproducibility of the CLEAN scores in four breast cancer datasets and five clustering algorithms described in the previous section (since Pearson's correlation implicitly re-scales data, only the Pearson's correlation clustering with re-scaled data was used in this case). The heatmap in Figure 4 represents the similarities of clusterings for different algorithms and different datasets in terms of the CLEAN and the cwCLEAN scores. Three groupings of clusterings-by-score type combinations clearly emerge: clusterings formed using Euclidean distances and un-scaled data, cwCLEAN scores for clusterings based on the re-scaled data and CSIMM algorithm using un-scaled data, and CLEAN scores based on the re-scaled data and CSIMM algorithm using un-scaled data.

**Figure 5:**
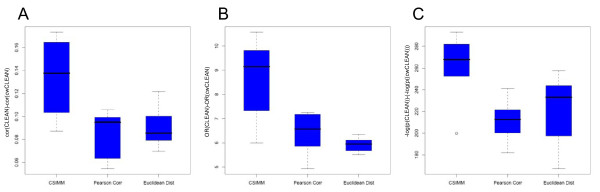
**Differences in the reproducibility of CLEAN and cwCLEAN scores**. Improvements in the reproducibility of CLEAN over cwCLEAN scores were demonstrated by box plots of differences in correlation coefficients, and odds ratios and *p*-values in 2-by-2 contingency tables of statistically significant scores. A) Box plots of differences in correlations between CLEAN and cwCLEAN scores of all 6 pairs of breast cancer datasets for three different clustering algorithms. Since all differences are positive, this indicates that the correlation coefficient was higher for CLEAN scores in each of the 6 pairs. B) Box plots of differences in odds ratios for 2-by-2 contingency tables of statistically significant CLEAN and cwCLEAN scores for all 6 pairs of breast cancer datasets and three different clustering algorithms. All differences are positive implicating higher reproducibility of CLEAN scores. C) Box plots of differences in the statistical significances in (-log_10_(*p*-values)) in the Fisher's Exact test for the same contingency tables as in B). The fact that all differences are positive again implicates higher reproducibility of CLEAN scores.

The improvement in reproducibility was further assessed by analyzing differences in correlations between CLEAN and cwCLEAN scores of all 6 pairs of breast cancer datasets for three different clustering algorithms (Figure 5A). Since all differences are positive, this indicates that the correlation coefficient was higher for CLEAN scores in each of the 6 pairs for all three algorithms. The increased reproducibility was also evident in the analysis utilizing the statistical significance cut-offs established by randomizing the gene labels for each clustering separately. For each pair of datasets we constructed a 2-by-2 contingency table based on the statistical significance scores (as in Table 1), and calculated differences in the odds ratios and the statistical significance of overlaps between lists of statistically significant genes in different datasets for a given algorithm and functional coherence score (CLEAN or cwCLEAN) (Figure 5B). All differences in odds ratios were positive implicating again higher reproducibility of CLEAN scores. Similarly, differences in the statistical significances (-log_10_(*p*-values)) of the Fisher's Exact tests for the same contingency tables were also all positive implicating the higher reproducibility of CLEAN scores (Figure 5C).

**Table 1:** Contingency table of genes with significant and non-significant CLEAN score in human and mouse tissues.

		Human CLEAN score	
		
		Significant (> 2.7)	Non-significant (< 2.7)
		
Mouse CLEAN score	Significant (> 3.2)	2,057	1,173
	
	Non-significant (< 3.2)	2,222	4,835

This table shows results for the gene-specific CLEAN score. The odds ratio is 3.82 and the Fisher Exact test *p*-value is 4.4 × 10^-207^.

We repeated a similar type of analysis for a mouse and human datasets profiling gene expression in different tissues (79 human and 61 mouse tissue types). [32]. After matching human and mouse probes using HomoloGene identifiers [33] we obtained 10,287 common genes that were represent on both microarray platforms. We constructed CSIMM-based gene clusterings for both species and applied CLEAN separately for the human and mouse datasets using both GO and KEGG based functional categories. Statistically significant correlation between the genes with statistically significant scores, using the Fisher's exact test for two-by-two tables, was firmly established for both CLEAN and cwCLEAN scores (Table 1 and 2 respectively). However, the statistical significance and the strength of association was considerably higher for the CLEAN score (odds ratio = 3.82 and *p*-value = 4.4 × 10^-207^) than for the cwCLEAN score (odds ratio = 1.49 and *p*-value = 1.8 × 10^-17^).

**Table 2:** Contingency table of genes with significant and non-significant cwCLEAN score in human and mouse tissues.

		Human cwCLEAN score	
		
		Significant(> 2.7)	Non-significant (< 2.7)
		
Mouse cwCLEAN score	Significant (> 3.2)	4,852	1,315
	
	Non-significant (< 3.2)	2,937	1,183

When using the cluster-wide cwCLEAN the odds ratio is 1.49 and the Fisher *p*-value is 1.8 × 10^-17^

### Unsupervised selection of informative genes

Reproducibly identifying genes whose expression patterns can delineate biologically meaningful groups of samples has been an important problem in computational biomedicine. We focus on the situation when the identity of samples belonging to different groups or even the number of the groups is not known in advance. In this case, the informative genes have to be selected in an unsupervised fashion. By studying the problem of classifying samples from different tissue types in the integrated mouse-human dataset, we demonstrate the utility of using the CLEAN score to select informative genes. We first identify genes with statistically significant CLEAN scores in mouse and human tissue expression profiling datasets. Then we show that expression profiles of these genes facilitate better separation of samples from different tissue types than expression profiles of genes not having statistically significant CLEAN scores. Furthermore, we demonstrate that the improvements in precision are significantly larger when using the CLEAN score than when using the cluster-wide cwCLEAN scores.

We created a total of 6 different gene lists and assessed their abilities to distinguish different tissue types in the combined human-mouse expression dataset. Gene lists were as follows:

#### All Genes

All 10,287 genes present in both microarray platforms.

#### Significant CLEAN score

Genes with significant CLEAN scores in both human and mouse datasets.

#### Non-significant CLEAN score

Genes with not significant CLEAN scores in both human and mouse datasets.

#### Significant cluster score

Genes with significant cwCLEAN scores in both human and mouse datasets.

#### Non-significant cluster score

Genes with not significant cwCLEAN scores in both human and mouse datasets.

#### COPA genes

Genes identified by applying the Cancer Outlier Profiler Analysis (COPA) [34] analysis. This COPA list of 2,668 genes was generated by performing COPA separately for human and mouse datasets, selecting the top 5,000 genes, and using the overlapping genes in the two datasets. This procedure was tuned to produce a number of genes that is similar to the number of genes with significant CLEAN scores.

**Figure 6:**
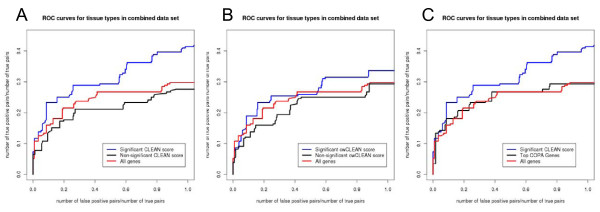
**Unsupervised selection of informative genes**. Genes were clustered based on their expression across different tissue samples and functional coherence scores are calculated for the human and mouse datasets separately. Ability of different groups of genes to facilitate correct grouping of samples from the same tissue type in the combined human-mouse dataset was assessed by constructing ROC curves. The ROC curve for clustering samples based on all 10,287 genes is inserted in each plot (red line) for the reference. A) ROC curves for clustering samples based on genes with the statistically significant CLEAN scores in both mouse and human datasets, and genes not statistically significant in either of the datasets. B) Same as A) for the cwCLEAN instead of CLEAN scores. C) ROC curves based on genes selected using COPA. The number of selected genes was identical to the number of genes with statistically significant CLEAN scores used in A).

Tissue samples were then clustered based on each of these gene lists using Euclidean distance, average linkage hierarchical clustering. Co-clustered pairs of samples derived from the same tissue type (regardless of whether they are human or mouse derived) were considered true positives, and co-clustered pairs of samples derived from different tissues were considered false positives. By cutting the hierarchical tree structure at all possible levels and each time recording the number of true and false positives we determined the receiver operating characteristic (ROC) for each of the gene lists. Since the number of positive pairs (232) is small compared to the number of negative pairs (38,828), we used the ratio of number of false positive pairs divided by the total number of positive pairs, as recently described [35], instead of the traditional false positive rates on the x-axis. Genes with significant CLEAN scores in both human and mouse tissue expression sets were significantly better in separating different tissue types than the genes with non-significant CLEAN scores (Figure 6A). Genes with significant cwCLEAN scores were marginally better in separating different tissue types than genes with non-significant cwCLEAN scores (Figure 6B), but the difference was considerably smaller than for the CLEAN score. Using COPA for selecting informative genes was completely ineffective as it did not show any improvement over using all genes (Figure 6C).

### Computational Infrastructure

We developed an open-source R package [36] that performs Clustering Enrichment Analysis (CLEAN). Typically, the user will provide a gene expression data set and a clustering of the genes. The package is intended for hierarchical clusterings but can also accommodate non-hierarchical clusterings such as *k*-means [4]. The package is compatible with a number of common input formats. GO and KEGG functional categories are derived from respective Bioconductor packages [37], and users can provide their own functional categories. The CLEAN package provides functions to compute the CLEAN score and generate output files to interactively display expression data together with gene and sample clusterings, and functional cluster annotation.

**Figure 7:**
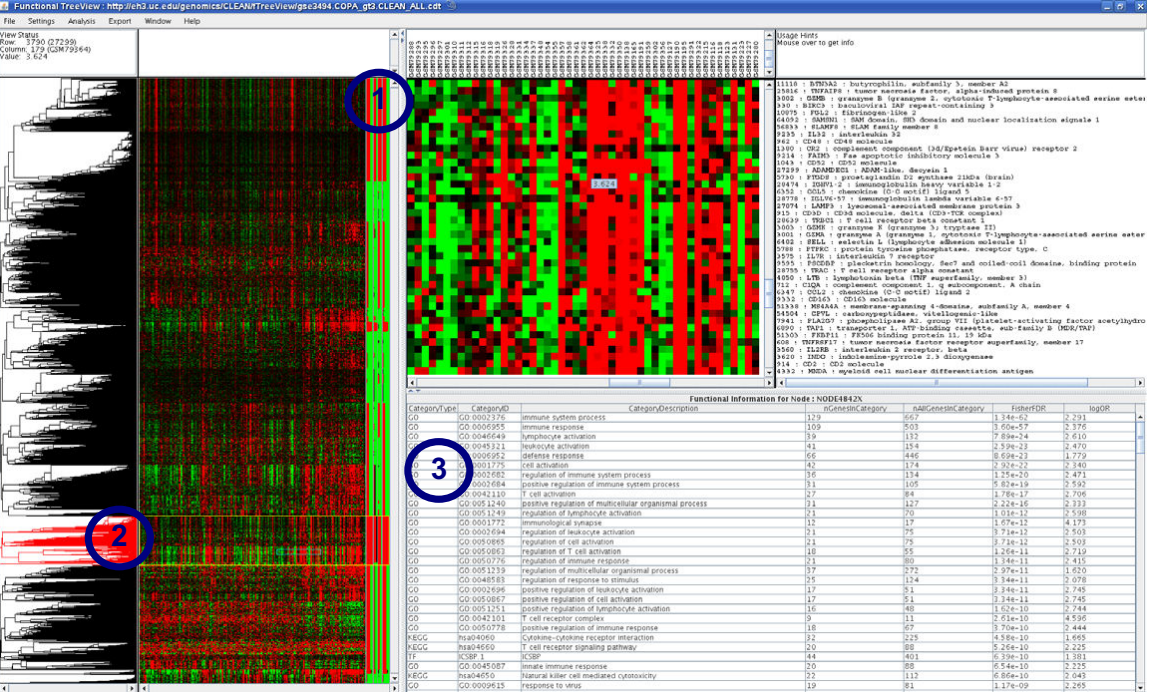
**Integrated software package**. CLEAN was implemented as an add-on R package [36]. The package integrates routines for calculating gene specific functional coherence scores and the interactive Java-based viewer Functional TreeView (FTreeView). The figure shows a screenshot of the fTreeView session displaying CLEAN results for one breast cancer dataset GSE3494 [28]. fTreeView was developed from the original Java TreeView [38] by adding panel 3, which displays functional cluster annotations generated by the CLEAN R package. This functionality enables seamless integration and browsing of functional categories associated with each cluster of genes (panel 2), which in turn can be selected based on the functional coherence scores (panel 1). The selected cluster of genes (panel 2) which we identified based on the overall high CLEAN scores (panel 1) is highly enriched for genes associate with immunity related Gene Ontology terms (FDR < 10^-60^) as well as two KEGG pathways, and putative targets of the Interferon Consensus Sequence-binding protein (ICSBP) transcription factor. These Results can be viewed interactively at  using the Java web-start version of FTreeView.

In addition, we extended the Java-based expression data viewing software TreeView [38] to interactively display functional cluster annotations and the cwCLEAN scores produced by the CLEAN R package. Figure 7 shows a screenshot of the new viewer we named Functional TreeView (FTreeview) displaying CLEAN results for the breast cancer dataset GSE3494 [28]. Panel 1 displays the per-gene functional coherence scores for individual category types. The broader the red bars are the higher is the score. Green indicates statistically non-significant functional coherence scores. Guided by the display of the CLEAN scores, the user can choose a subset of genes by selecting a branch of the hierarchical gene clustering tree (panel 2). Functional cluster annotations generated by CLEAN for the selected group of genes displayed in panel 3. The interactive display of functional annotations is the major new feature of FTreeView, and it allows for seamless integration and browsing of functional categories associated with each cluster of genes. Such an integrated view of clustering results, expression patterns and the enriched functional categories, facilitates a straightforward interactive identification of functionally coherent patterns of expression. For example, the selected cluster of genes (panel 3) which we identified based on the overall high CLEAN scores (panel 1) is highly enriched for genes associate with immunity related Gene Ontology terms (FDR < 10^-60^) as well as two KEGG pathways, and putative targets of the Interferon Consensus Sequence-binding protein (ICSBP) transcription factor. FTreeView is available as a stand-alone or as a Web Start application from our server .

## Discussion

Integrating biological knowledge encoded in lists of functionally related genes into the analysis of genome-wide functional genomics data is an increasingly important aspect of analyzing genomics data. In the context of cluster analysis, such integration is necessary for selecting meaningful clusters of genes, and for the adequate biological interpretation of patterns defined by such clusters. We developed a computational framework for analytically and visually integrating knowledge-based functional categories with the cluster analysis of genomics data. The framework is based on the gene-specific functional coherence score derived by correlating the clustering structure as a whole with functional categories of interest. The statistical significance of coherence scores is established by comparing them to the empirical null-distribution obtained by randomly permuting gene identifiers.

We established the reproducibility of the CLEAN score across related gene expression datasets, and its utility in comparing the functional coherence of different clusterings and in unsupervised selection of genes that discriminate between biologically meaningful groups of samples. When compared to the commonly used cluster-wide assessment of functional coherence, the CLEAN score exhibits higher reproducibility across different microarray datasets. Genes selected based on the CLEAN score produced more precise sample groupings than genes selected using either cluster-wide score or by using COPA algorithm.

**Figure 8:**
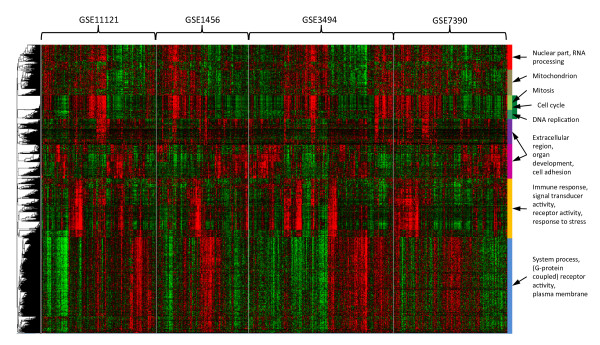
**Expression patterns of genes with statistically significant CLEAN scores in four independent breast cancer datasets**. The heatmap indicates that all genes belong to clusters with coherent expression patterns in each dataset. Functional categories on the right-hand side indicate the enriched functional categories for each global cluster of co-expressed genes. This heatmap can be interactively browsed using FTreeView at .

It is important notice that by using the CLEAN score instead of the traditional cluster-wide approach one cannot use the guilt-by-association principle [39] to hypothesize the function of non-annotated genes. Our analysis of the four breast cancer datasets yielded one obvious example of a relevant gene (FOXM1) with a high cwCLEAN score and the CLEAN score of zero in all four breast cancer datasets. FOXM1 is a proliferation-associated transcription factor [40] which has recently been clearly implicated to be an important regulator in the cell cycle progression [41]. However, functional annotations for this gene (Gene Ontologies and KEGG) do not reflect these recent findings and consequently FOXM1 was not associated with the "cell cycle" cluster based on the CLEAN score (Figure 8).

One way to think about the differences between CLEAN and cwCLEAN score in terms of differences between assuming functional coherence based on co-clustering (guilt-by-association, cwCLEAN) vs. having some additional pre-existing evidence of functional relationship (CLEAN). Our results in this context implicate that in the case of breast cancer and tissue datasets the previous evidence of functional relationship is overall more reliable than guilt-by-associations relationships arising only from the cluster analysis on their own. It is possible that in some other situations, new functional relationships would dominate the existing one and the opposite would be the case. Calculating the difference between the two scores can quickly implicate novel functional relationships arising from the data analysis alone.

A systematically different approach to integrating the experimental data and prior knowledge is to incorporate the functional information into the clustering algorithm itself [42-46]. While conceptually appealing, such methods have a more limited applicability than the framework presented here and have not been widely used. Our framework follows the commonly utilized post-hoc integration approach in which cluster analysis is performed first using the experimental data and integration is achieved in the post-hoc analysis. The ability to validate the clusters produced by analyzing experimental data, and the transparency about how exactly the different types of information is utilized in constructing clusters is most likely the reason for the popularity and the wide usage of post-hoc approaches. When the functional knowledge is used in the process of constructing clusters, it can no longer be employed to provide the guidance in selecting biologically meaningful clusters.

## Conclusion

We directly demonstrate that integrating prior biological knowledge encoding in the lists of functionally coherent genes improves the reproducibility of clustering results. We also demonstrate that our gene-specific functional coherence score, which differentiates between the levels of functional coherence for genes within the same cluster, shows higher reproducibility than the cluster-wide score. The CLEAN score also produced more informative genes for distinguishing different sample types than the cluster-wide score. This implicates that the gene-specificity of the CLEAN score is a fundamentally different and, at least in some circumstances, better approach for integrating biological knowledge with results of the cluster analysis than previously used cluster-based scores.

## Methods

### Data Preprocessing, Gene Selection and Clustering

Raw data files (Affymetrix HG-U133A CEL files) of four independent human breast cancer datasets (GEO expression series GSE1456 [29], GSE3494 [28], GSE7390 [31], and GSE11121 [30]) were downloaded from the public repository GEO [33]. Each dataset was RMA-preprocessed [47] separately using the Entrez Gene-based custom CDF (version 10) from the Psychiatry/MBNI Microarray Lab at the University of Michigan ('Brainarray') [48]. Preprocessed data files of a large-scale tissue expression data set [32] were also downloaded from the same repository. The tissues included both human (GEO dataset record GDS596) and mouse (GDS592). For genes with multiple probes per Entrez gene ID, in each case, the probeset with the highest median expression value per probeset was selected as the representative probeset for that gene. To match corresponding genes across species, the HomoloGene database [33] was used.

We applied a mild variation filter using Cancer Outlier Profiler Analysis (COPA, 95^th ^percentile) [34] to select the top 10,000 genes to be clustered in each of the human breast cancer datasets (GSE1456, GSE3494, GSE7390, GSE11121). In each dataset expression values were centered by setting the median value of each gene to zero (subtracting the gene-specific medians) and clustering analyses were performed for each dataset and species independently using hierarchical clustering with three different similarity metrics or distance metrics, respectively:

• Context-Specific Infinite Mixture Models (CSIMM) [22]. For any given pair or genes, this Bayesian method estimates the posterior pairwise probability (PPP) of the genes being co-clustered. The resulting PPP matrix is used as the similarity measure for the hierarchical clustering algorithm.

• Pearson Correlation of gene expression values as the similarity measure.

• Euclidian Distance based on per-gene normalized expression values as the distance measure.

All three hierarchical clustering algorithms used Average Linkage.

Each clustering analysis was then repeated after further variance based re-scaling each dataset by dividing expression levels by their standard deviation for each gene and each datasets separately. When computing the Pearson correlation, expression values are implicitly divided by the standard deviation. Thus, this additional normalization step did not significantly affect Pearson's correlations.

All statistical analyses were performed using the statistical programming environment R version 2.7.1 [36] and Bioconductor release 2.2 [37].

### Clustering Enrichment Analysis

Clustering Enrichment Analysis (CLEAN) is based on testing every possible cluster within a gene clustering for statistically significant enrichment of biological categories. A background gene list (e.g. all genes represented on the microarray) is given as well as a hierarchical clustering of some or all genes in the background list. The method was implemented as described in Algorithm 1.

**Algorithm 1**. Clustering Enrichment Analysis (CLEAN).

1. Define one or more sets of biological categories with sufficient representation in the background gene list

2. Determine all possible gene clusters within a given size range

3. For each gene cluster

3.1 For each functional category, determine the 2 × 2 contingency table and perform Fisher's Exact test

3.2 Compute *q*-values, that is the adjusted Fisher *p*-values, to account for multiple testing

3.3 Record categories the cluster is significantly enriched with and corresponding *q*-values

4. Compute the cluster-wide cwCLEAN score

4.1 Determine the minimum *q*-value for each gene cluster

4.2 Sort gene clusters by minimum *q*-value starting with the lowest (i.e. most significant) *q*-value.

4.3 Prune cluster supersets with less significant *q*-value to avoid 'spill-over' effect, i.e. remove gene clusters whose significant functional enrichment score is likely due to a single subtree.

4.4 For each gene, find the minimum *q*-value over *all remaining clusters *the gene is member of

4.5 For each gene, find the minimum *q*-value over all category sets (e.g. GO and KEGG)

4.6 The cwCLEAN is the resulting -log_10_-transformed minimum per-gene *q*-value.

5. Compute the gene-specific CLEAN score

5.1 For each gene, find the minimum *q*-value over *all clusters and all categories *the gene is member of.

5.2 For each gene, find the minimum *q*-value over all category sets (e.g. GO and KEGG)

5.3 The CLEAN is the resulting -log_10_-transformed minimum per-gene *q*-value.

#### Defining Functional Categories

A functional category is defined as a non-empty set of genes representing a biological concept such as "cell cycle", "immunological synapse" or "cytokine-cytokine receptor activation". The method is designed to accommodate any set of functional categories such that each category is comprised of a list of genes that has user-specified minimum overlap with the background gene list. Here, sets of categories were either downloaded from publically accessible databases such as Gene Ontology (GO) [24], Kyoto Encyclopedia of Genes and Genomes (KEGG) [25,26], or were defined based on the Transfac database [27].

More specifically, functional categories based on GO and KEGG were downloaded as R packages [36,37] while co-regulation based categories (CG) were derived computationally. Transcription factor and corresponding gene promoter data [27] and DNA sequence data [49] was downloaded. For each of the 304 human transcription factors with at least one position-weight matrix (PWM) in the Transfac version 12.1 database, a score was computed for each of the 24,190 genes, as to how likely they were to have a corresponding binding motif within 1 kbp of their transcriptional start site. The respective 750 top-scoring genes (or fewer if the score was not significant for at least 750 genes) were assigned to each transcription factor to define the respective functional categories.

For compatibility, all gene identifiers were converted to Entrez gene IDs, and matched across species where necessary using the HomoloGene database [33]. Subsequent analyses were restricted to categories that had at least ten genes in common with the respective background gene list (e.g. the genes represented on the microarray platform).

#### Obtaining All Possible Gene Clusters

Given a hierarchical gene tree, a list of all possible gene clusters is obtained by recursively traversing the tree structure and at each node recording the list of corresponding genes. The size of clusters is limited to a user-specified range. Here, clusters smaller than 10 genes and larger than 1,000 were disregarded.

#### Determining significant functional enrichment

To determine whether a functional category is over-represented in a given gene cluster, i.e. more genes of the functional category are present in the cluster than expected by chance, a two-by-two contingency table (number of genes in the cluster and category, in the cluster and not in the category, etc.) is constructed and Fisher's Exact Test is performed. The procedure is repeated for each category within a category set (e.g. "GO", "KEGG") and *q*-values (i.e. adjusted *p*-values) are computed to control the false discovery rate (FDR) [50]. Categories with a *q*-value not greater than a user-defined cutoff are considered significant. The default *q*-value cutoff is 0.1.

#### Procedure for Non-hierarchical Methods

The procedure can be extended to non-hierarchical methods such as *k*-means [4]. For a fixed number *k *of clusters, a mutually exclusive set of gene clusters is already given and step 2 in Algorithm 1 is skipped. If the user specifies a range *K *of cluster numbers, step 2 in Algorithm 1 is preceded by Algorithm 2 which generates a hierarchical gene clustering as a means to average over multiple runs of the non-hierarchical clustering algorithm.

**Algorithm 2**. Averaging gene clusterings over *n *runs of a non-hierarchical clustering algorithm

1. For each *k *in *K*, run the non-hierarchical clustering algorithm

2. For each gene *i*

2.1 For each gene *j *≠ *i*, count the number *n*_*ij *_of clusterings where *i *and *j *are in the same cluster

2.2 For each gene *j *≠ *i*, compute *p*_*ij *_= *n*_*ij*_/*n*, where *n *is the size of *K*

3. Use the *p*_*ij *_as a similarity metric and average linkage as a summary method to generate a hierarchical clustering.

### R package and FTreeView tool

An R package to perform CLEAN and an open-source Java tool to interactively display gene expression data, gene and sample clustering, gene annotation, and functional cluster annotation can be freely downloaded from the authors' web-site .

## Abbreviations

CG: co-regulation groups; ChIP: Chromatin Immunoprecipitation; CLEAN: Clustering Enrichment Analysis; COPA: Cancer Outlier Profiler Analysis; CSIMM: Context-Specific Infinite Mixture Models; cwCLEAN: cluster-wide CLEAN score; FDR: false discovery rate; GO: Gene Ontology; KEGG: Kyoto Encyclopedia of Genes and Genomes; OR: odds ratio; PPP: posterior pairwise probability; PWM: position-weight matrix; RMA: Robust Multichip Average; ROC: Receiver Operating Characteristic.

## Authors' contributions

JF developed the methods and the R package, performed all analyses, interpreted results and drafted the manuscript. MM conceived the methodology and provided guidance in the development, design, analysis, interpretation of results, and drafting of the manuscript. VJ developed FTreeView and ZH constructed some of the functional categories used in the analysis.
